# Causal association of gut microbiota on spondyloarthritis and its subtypes: a Mendelian randomization analysis

**DOI:** 10.3389/fimmu.2024.1284466

**Published:** 2024-02-08

**Authors:** Jun Tang, Shiyan Mo, Lina Fan, Shihui Fu, Xiaofei Liu

**Affiliations:** ^1^ Experimental Teaching Management Center, Chongqing Medical University, Chongqing, China; ^2^ Department of Rheumatology and Immunology, Hainan Hospital of Chinese People's Liberation Army of China (PLA) General Hospital, Sanya, Hainan, China; ^3^ Department of Cardiovascular, Hainan Hospital of Chinese People’s Liberation Army of China (PLA) General Hospital, Sanya, Hainan, China

**Keywords:** spondyloarthritis, gut microbiota, Mendelian randomization, causal effect, genome wide association study

## Abstract

**Background:**

Despite establishing an association between gut microbiota and spondyloarthritis (SpA) subtypes, the causal relationship between them remains unclear.

**Methods:**

Gut microbiota data were obtained from the MiBioGen collaboration, and SpA genome-wide association study (GWAS) summary data were obtained from the FinnGen collaboration. We conducted a two-sample Mendelian randomization (MR) analysis using the inverse-variance-weighted method supplemented with four additional MR methods (MR-Egger, weighted median, simple mode, and weighted mode). Pleiotropy and heterogeneity were also assessed. Reverse MR analysis was used to detect reverse causal relationships.

**Results:**

We identified 23 causal links between specific gut microbiota taxa and SpA levels. Of these, 22 displayed nominal causal associations, and only one demonstrated a robust causal connection. Actinobacteria id.419 increased the risk of ankylosing spondylitis (AS) (odds ratio (OR) = 1.86 (95% confidence interval (CI): 1.29–2.69); *p* = 8.63*E*−04). The family Rikenellaceae id.967 was associated with a reduced risk of both AS (OR = 0.66 (95% CI: 0.47–0.93); *p* = 1.81*E*−02) and psoriatic arthritis (OR = 0.70 (95% CI: 0.50–0.97); *p* = 3.00*E*−02). Bacillales id.1674 increased the risk of AS (OR = 1.23 (95% CI: 1.00–1.51); *p* = 4.94*E*−02) and decreased the risk of enteropathic arthritis (OR = 0.56 (95% CI: 0.35–0.88); *p* = 1.14*E*−02). Directional pleiotropy, or heterogeneity, was not observed. No reverse causal associations were observed between the diseases and the gut microbiota.

**Conclusion:**

Our MR analysis suggested a genetic-level causal relationship between specific gut microbiota and SpA, providing insights into the underlying mechanisms behind SpA development mediated by gut microbiota.

## 1 Introduction

Spondyloarthritis (SpA) is a group of rheumatic diseases characterized primarily by sacroiliitis and accompanied by musculoskeletal and extra-articular manifestations. It encompasses several subtypes, including ankylosing spondylitis (AS), enteropathic arthritis (EA), psoriatic arthritis (PsA), reactive arthritis, undifferentiated SpA, and juvenile SpA (1).

The aetiology and pathogenesis of SpA remain unknown, with substantial evidence indicating a connection between intestinal and articular inflammation (2–4). The investigation of the gut-joint axis has become a significant domain in unraveling the pathogenesis of SpA (5). However, studies investigating gut microbiota dysbiosis in patients with SpA have produced inconsistent results, and the key bacteria identified vary between studies (6–15). Previous studies have failed to elucidate the causal association between gut microbiota and the development of SpA (16, 17), necessitating further investigation.

Mendelian randomization (MR) is a method utilized to infer causal associations between instrumental variables, typically single-nucleotide polymorphisms (SNPs) (18). By utilizing natural genetic variations associated with a specific exposure factor, MR simulates the effects of randomized controlled trials and infers the causal impact of the exposure factor on a specific outcome (19). SNPs are randomly assigned prior to the occurrence of disease, thereby enabling the elimination of potential confounding factors and reverse causality influences (20). A recent study using MR examined the potential causal association between six bacteria and AS, but no significant causal associations were found (21). However, considering the growing evidence supporting the significant role of the gut microbiota in the development of SpA, there is still no consensus regarding the specific taxonomic group of gut microbiota that has the greatest impact on SpA. Additionally, it remains unclear whether there is a mutual influence of gut bacteria among different subtypes of SpA, highlighting the need for further research to clarify this aspect.

Our research focuses on investigating the causal association between the gut microbiota and three types of SpA: AS, PsA, and EA. Another objective is to identify shared bacteria among these three diseases. This approach identifies gut bacteria that have a causal relationship with SpA, providing new avenues for future prevention and treatment of SpA.

## 2 Methods

### 2.1 Study design

**Figure 1:**
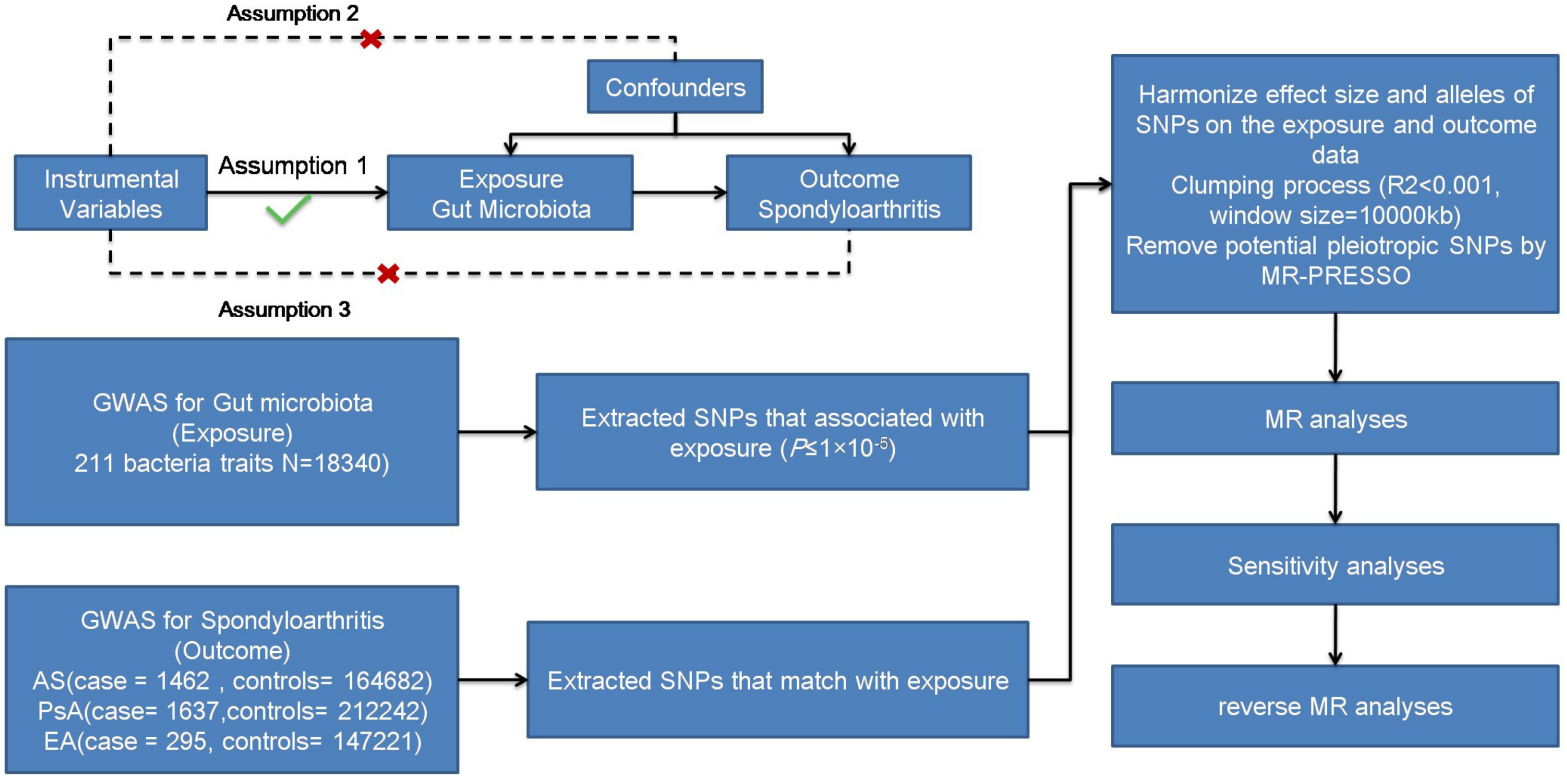
Three assumptions of MR and process diagram.

We aimed to examine the causal association between gut microbiota and different subtypes of SpA (AS, PsA, and EA) using a two-sample MR approach. **Figure 1** illustrates the schematic diagram of the study design. MR studies must adhere to three key assumptions for reliable and valid results. Firstly, the first assumption is that genetic variants are used as instrumental variables for the exposure of interest. These instrumental variables should be strongly associated with the exposure but not directly associated with confounding factors or the outcome. Secondly, the instrumental variables should be independent of confounding factors that could bias the causal relationship. Lastly, the outcome variable should be solely associated with the exposure factor, without direct influence from other variables.

### 2.2 Data sources

Genome-wide association study (GWAS) data concerning human gut microbiota, which served as the exposure variable, were obtained from the MiBioGen study. This study involved a total of 18,340 individuals from 24 cohorts and recorded 211 taxonomic units (131 genera, 35 families, 20 orders, 16 phyla, and nine classes), as well as 122,110 SNPs (22). GWAS summary data for the different subtypes of SpA (AS, PsA, and EA) were obtained from the FinnGen Biobank. Patients with AS (21), PsA (23), and EA were selected using ICD-10 diagnostic codes. **Table 1** provides detailed data. Ethical approval is not required for this study as all summary-level data have already obtained ethical approval in previous GWAS studies.

**Table 1:** GWAS data used in this study.

Trait	Consortium	Case	Control	SNPs	Year
Gut microbiota	MiBioGen	18,340			
Ankylosing spondylitis	FinnGen Biobank	1,462	164,682	16,380,022	2021
Arthropathic psoriasis	FinnGen Biobank	1,637	212,242	16,380,462	2021
Enteropathic arthropathies	FinnGen Biobank	295	147,221	16,380,134	2021

GWAS, genome-wide association studies; SNP, single-nucleotide polymorphism.

### 2.3 Genetic instruments selection

To ensure robustness and accuracy while adhering to the assumptions necessary for MR analysis, a quality check was performed on the SNPs to obtain valid instruments (**Figure 1**). Due to the strict threshold (*p* < 5.0 × 10^−8^), only a few instrumental variables were obtained. To increase the number of instrumental variables, a threshold of *p* < 1.0 ×10^−5^ was adopted (24, 25). Palindromic SNPs and SNPs not present in the outcomes (AS, PsA, and EA) were excluded from the instrumental variables. We utilized the PhenoScanner database (http://www.phenoscanner.medschl.cam.ac.uk/phenoscanner, accessed on 7 October 2022) to exclude confounding factors reported in the literature, including body mass index (BMI) and smoking (26). Linkage disequilibrium was eliminated using a threshold of *r* ^2^ < 0.001 and a clumping distance of 10,000 kb. The basic formula for the *F*-statistic in MR analysis is: *F* = (*r* ^2^/(1−*r* ^2^)) * ((*N*−*k*−1)/*k*), where *r* ^2^ is the proportion of variance in the exposure variable explained by the instrumental variables, *N* is the sample size, and *k* is the number of instrumental variables used in the analysis. The *F*-statistic was calculated for all the instrumental variables, with a value of > 10 considered sufficient and unbiased (27, 28).

### 2.4 MR analysis

MR analysis employed five methods, including inverse variance weighting (IVW), MR-Egger, weighted median, simple mode, and weighted mode, to infer causal relationships (29–31). IVW was the primary method, with the other four serving as supplementary approaches. Estimates were presented using odds ratios (ORs) along with their respective 95% confidence intervals (CIs). The study also investigated reverse causality, with SpA considered the exposure and gut microbiota the outcome. The procedures for the reverse and regular MR analyses were identical.

### 2.5 Sensitivity analysis

Heterogeneity in IVW and MR Egger was detected by calculating Cochran’s *Q* statistic and its corresponding *p*-value. The evaluation of pleiotropy, a potential source of heterogeneity, was performed using the MR-Egger intercept test. The leave-one-out analysis method was utilized to identify and eliminate potential outliers.

### 2.6 Statistical analysis

To ensure a more rigorous interpretation of the causal relationship between bacterial species and health outcomes, the Bonferroni correction was applied to address the issue of increased false-positive results when conducting multiple hypothesis tests for different levels of bacterial classification. In our study, since statistical tests were conducted for 131 genera, 35 families, 20 orders, 16 classes, and nine phyla, the significance level was adjusted to 0.05/131 (3.8 × 10^−4^) for genera, 0.05/35 (1.4 × 10^−3^) for families, 0.05/20 (2.5 × 10^−3^) for orders, 0.05/16 (3.1 × 10^−3^) for classes, and 0.05/9 (5.5 × 10^−3^) for phyla after applying the Bonferroni correction (32). MR results with *p*-values lower than the adjusted significance level were considered statistically significant, while MR estimates with *p*-values less than 0.05 were considered nominally significant (31). All analyses were conducted using the TwoSample MR package (version 0.5.6) in R 4.0.2 (31, 33).

## 3 Results

### 3.1 Selection of instrumental variables

Overall, 1,784 unique SNPs were identified as the final instrumental variables, which were associated with exposure at *p*-values < 1*E*−5 (**Supplementary Table S1**). They were classified into five different levels of bacterial classification. There were 54 instrumental variables in phyla, 181 instrumental variables in classes, 14 instrumental variables in orders, 313 instrumental variables in families, and 1,222 instrumental variables in genera. The *F*-statistics range from 16.91 to 58.15, and all the *F*-values are greater than 10.

### 3.2 Mendelian randomization analysis

**Figure 2:**
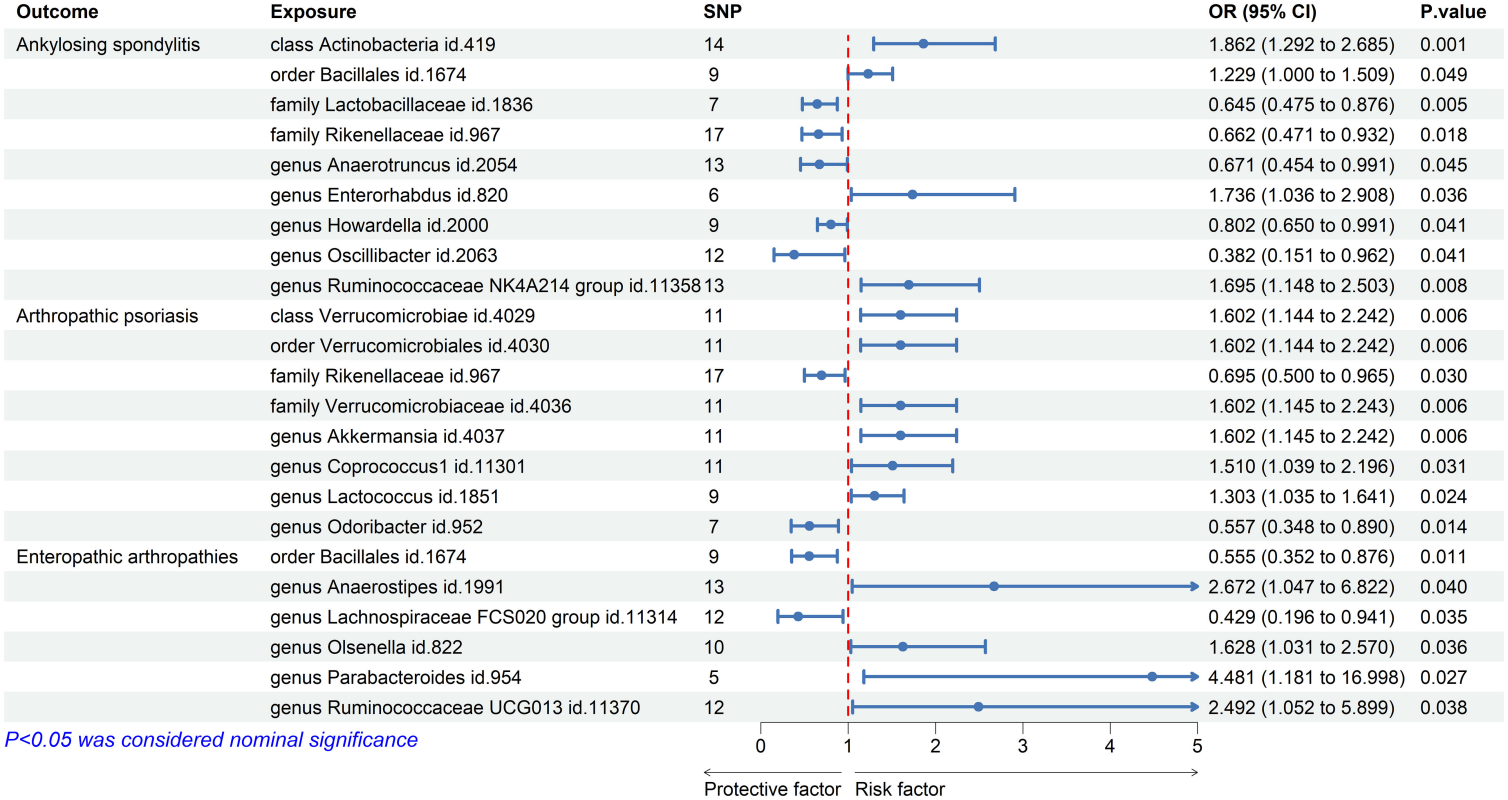
Forest plot of gut microbiota taxa associated with spondyloarthritis by inverse variance weighting.

Using the IVW method, we discovered significant associations between the nine microbial taxa and AS. The results indicate that the class Actinobacteria id.419, order Bacillales id.1674, genus Enterorhabdus id.820, and genus Ruminococcaceae NK4A214 group id.11358 may increase the risk of AS. By contrast, the families Lactobacillaceae id.1836 and Rikenellaceae id.967, and the genera Anaerotruncus id.2054, Howardella id.2000, and Oscillibacter id.2063 were negatively associated with AS (**Figure 2**). **Supplementary Table S2** displays the IVW results as well as the results using the four complementary methods (MR-Egger, weighted median, simple mode, and weighted mode). After using Bonferroni correction, the Actinobacteria id.419 class (OR = 1.86 (95% CI: 1.29–2.69); *p* = 8.63*E*−04) remained a risk factor for AS. In the reverse MR analysis, no causal association was found between AS exposure variable and gut microbiota as an outcome (**Supplementary Table S3**).

The results of IVW showed that the class Verrucomicrobia id.4029, the order Verrucomicrobiales id.4030, the family Verrucomicrobiaceae id.4036, and the genera Akkermansia id.4037, Coprococcus1 id.11301, and Lactococcus id.1851 were associated with an increased risk of PsA, whereas the family Rikenellaceae id.967 and the genus Odoribacter id.952 were associated with a decreased risk of PsA (**Figure 2**; **Supplementary Table S2**). However, after Bonferroni correction, these microbial taxa did not show any significant causal effect on PsA. In the reverse MR analysis, no causal association was found between PsA as an exposure variable and gut microbiota as an outcome (**Supplementary Table S3**).

We also used the IVW method to evaluate the causal associations between gut microbiota and EA. The results indicated that the genera Anaerostipes id.1991, Olsenella id.822, Parabacteroides id.954, and Ruminococcaceae UCG013 id.11370 were associated with an increased risk of EA, whereas the order Bacillales id.1674 and the genus Lachnospiraceae FCS020 group id.11314 were associated with a decreased risk (**Figure 2**; **Supplementary Table S2**). However, after Bonferroni correction, these microbial taxa did not show significant causal effects on EA. In the reverse MR analysis, the genera Parabacteroides id.954 and Lachnospiraceae FCS020 showed nominal reverse causal relationships with EA; however, these associations disappeared after Bonferroni correction.

The MR results are also presented as scatterplots, and the causal associations of gut microbiota on the risks of AS (**Supplementary Figure S1**), PsA (**Supplementary Figure S2**), and EA (**Supplementary Figure S3**) are illustrated. **Supplementary Figures S4**-**S6** indicate the forest plot results for the bacteria in terms of AS, PsA, and EA, respectively.

**Figure 3:**
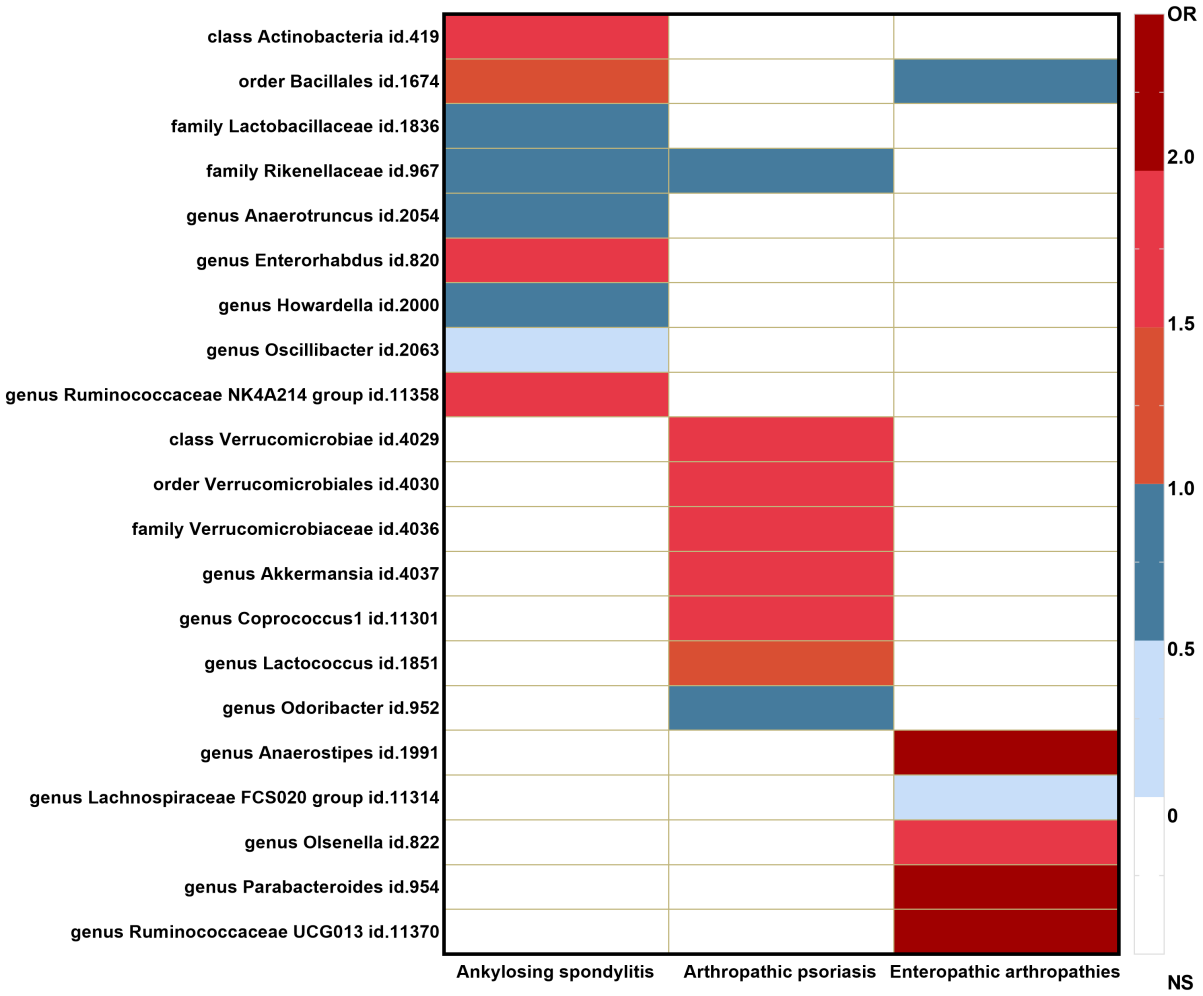
Heatmap of GM taxa causally associated with ankylosing spondylitis, psoriatic arthritis, and enteropathic arthritis.

The causal associations between the gut microbiota taxa identified in our study and the risks of AS, PsA, and EA were visualized using a heatmap (**Figure 3**). The Rikenellaceae id.967 family showed a reduced risk of both AS (OR = 0.66 (95% CI: 0.47–0.93); *p* = 1.81*E*−02) and PsA (OR = 0.70 (95% CI: 0.50–0.97); *p* = 3.00*E*−02), while the order Bacillales id.1674 increased the risk of AS (OR = 1.23 (95% CI: 1.00–1.51); *p* = 4.94*E*−02) and decreased the risk of EA (OR = 0.56 (95% CI: 0.35–0.88); *p* = 1.14*E*−02).

### 3.3 Sensitivity analysis

Cochran’s *Q* test was used to assess heterogeneity, and MR-Egger and IVW tests were conducted as well. Except for the genus Oscillibacter id.2063, which showed heterogeneity in the MR analysis, all other *p*-values were greater than 0.05 (**Table 2**).

**Table 2:** The heterogeneity results from the Cochran’s *Q* test.

Subtype	Bacterial taxa	MR-Egger			IVW		
		*Q*	*Q*_*df*	*p*-value	*Q*	*Q_df*	*p*-value
Ankylosing spondylitis	*Class Actinobacteria id.419*	11.12	12	5.19*E*−01	11.12	13	6.01*E*−01
	*Order Bacillales id.1674*	2.59	7	9.20*E*−01	4.42	8	8.18*E*−01
	*Family Lactobacillaceae id.1836*	2.38	5	7.95*E*−01	2.74	6	8.41*E*−01
	*Family Rikenellaceae id.967*	7.51	15	9.42*E*−01	7.51	16	9.62*E*−01
	*Genus Anaerotruncus id.2054*	8.08	11	7.06*E*−01	10.64	12	5.60*E*−01
	*Genus Enterorhabdus id.820*	5.35	4	2.53*E*−01	8.48	5	1.32*E*−01
	*Genus Howardella id.2000*	6.03	7	5.36*E*−01	6.44	8	5.98*E*−01
	*Genus Oscillibacter id.2063*	94.81	10	5.94*E*−16	113.16	11	4.27*E*−19
	*Genus Ruminococcaceae NK4A214 group id.11358*	10.86	11	4.55*E*−01	13.54	12	3.31*E*−01
Arthropathic psoriasis	*Class Verrucomicrobiae id.4029*	8.66	9	4.69*E*−01	8.99	10	5.33*E*−01
	*Order Verrucomicrobiales id.4030*	8.66	9	4.69*E*−01	8.99	10	5.33*E*−01
	*Family Rikenellaceae id.967*	10.96	15	7.56*E*−01	14.46	16	5.65*E*−01
	*Family Verrucomicrobiaceae id.4036*	8.66	9	4.69*E*−01	8.99	10	5.33*E*−01
	*Genus Akkermansia id.4037*	8.66	9	4.70*E*−01	8.98	10	5.34*E*−01
	*Genus Coprococcus1 id.11301*	9.15	9	4.24*E*−01	9.15	10	5.18*E*−01
	*Genus Lactococcus id.1851*	5.31	7	6.23*E*−01	5.91	8	6.57*E*−01
	*Genus Odoribacter id.952*	2.47	5	7.81*E*−01	5.00	6	5.43*E*−01
Enteropathic arthropathies	*Order Bacillales id.1674*	3.00	7	8.85*E*−01	6.62	8	5.78*E*−01
	*Genus Anaerostipes id.1991*	11.41	11	4.10*E*−01	12.90	12	3.76*E*−01
	*Genus Lachnospiraceae FCS020 group id.11314*	8.55	10	5.75*E*−01	8.70	11	6.49*E*−01
	*Genus Olsenella id.822*	0.60	8	1.00*E*+00	0.87	9	1.00*E*+00
	*Genus Parabacteroides id.954*	3.14	3	3.71*E*−01	3.32	4	5.05*E*−01
	*Genus Ruminococcaceae UCG013 id.11370*	10.71	10	3.81*E*−01	10.72	11	4.67*E*−01

IVW, inverse-variance weighted.

The Egger intercept was used to evaluate the horizontal pleiotropy, and the results showed no evidence of horizontal pleiotropy (**Table 3**). The leave-one-out results of the above bacteria for AS, PsA, and EA are shown in **Supplementary Figures S7**-**S9**, respectively.

**Table 3:** Pleiotropy results from Egger intercept analysis.

Subtype	Bacterial taxa	Egger_intercept	S.E.	*p*-value
Ankylosing spondylitis	*Class Actinobacteria id.419*	0.00	0.04	9.76*E*−01
	*Order Bacillales id.1674*	0.09	0.07	2.19*E*−01
	*Family Lactobacillaceae id.1836*	−0.03	0.06	5.76*E*−01
	*Family Rikenellaceae id.967*	0.00	0.04	9.66*E*−01
	*Genus Anaerotruncus id.2054*	0.06	0.04	1.38*E*−01
	*Genus Enterorhabdus id.820*	0.12	0.08	2.01*E*−01
	*Genus Howardella id.2000*	0.04	0.07	5.42*E*−01
	*Genus Oscillibacter id.2063*	−0.22	0.16	1.94*E*−01
	*Genus Ruminococcaceae NK4A214 group id.11358*	−0.07	0.05	1.30*E*−01
Arthropathic psoriasis	*Class Verrucomicrobiae id.4029*	0.03	0.05	5.78*E*−01
	*Order Verrucomicrobiales id.4030*	0.03	0.05	5.78*E*−01
	*Family Rikenellaceae id.967*	0.07	0.04	8.08*E*−02
	*Family Verrucomicrobiaceae id.4036*	0.03	0.05	5.80*E*−01
	*Genus Akkermansia id.4037*	0.03	0.05	5.82*E*−01
	*Genus Coprococcus1 id.11301*	0.00	0.03	9.59*E*−01
	*Genus Lactococcus id.1851*	0.05	0.07	4.63*E*−01
	*Genus Odoribacter id.952*	−0.09	0.06	1.72*E*−01
Enteropathic arthropathies	*Order Bacillales id.1674*	0.29	0.15	9.88*E*−02
	*Genus Anaerostipes id.1991*	0.13	0.11	2.55*E*−01
	*Genus Lachnospiraceae FCS020 group id.11314*	0.03	0.08	7.05*E*−01
	*Genus Olsenella id.822*	−0.05	0.10	6.15*E*−01
	*Genus Parabacteroides id.954*	0.16	0.38	7.04*E*−01
	*Genus Ruminococcaceae UCG013 id.11370*	−0.01	0.10	9.42*E*−01

S.E., standard error.

## 4 Discussion

Our study is the first to identify the causal effects of gut microbiota on various subtypes of SpA. Actinobacteria id.419 increased the risk of AS, while Rikenellaceae id.967 protected against both AS and PsA. Bacillales id.1674 increased the risk of AS but decreased the risk of EA. These findings shed light on the potential role of the gut microbiota in influencing different SpA subtypes.

Our study revealed a robust causal relationship wherein the Actinobacteria id.419 class remained significantly associated with an increased risk of AS, even after adjusting for multiple comparisons using Bonferroni correction. Patients with AS have a higher abundance of Actinobacteria in their gut compared to healthy individuals (10, 34). Another study proposed that Actinobacteria may regulate the ubiquitination of IκB-α, activate the NF-kB signaling pathway, and promote the accumulation of inflammatory factors, thereby contributing to the progression of AS (35). Our study further reveals a causal relationship between Actinobacteria and the onset of AS, suggesting that Actinobacteria may be implicated as a potential cause of AS.

In our investigation of the causal relationship between various SpA subtypes and gut bacteria, we discovered that the Rikenellaceae id.967 family exhibited a potential protective effect against AS and PsA, albeit with nominal significance. Notably, previous studies have reported an elevated abundance of Rikenellaceae in terminal ileum biopsy samples from patients with AS (36), as well as a positive correlation between psoriasis-like pathological features and Rikenellaceae (37). Based on these findings, we hypothesize that the increased presence of Rikenellaceae may offer some degree of protection against disease pathogenesis but may also coincide with heightened disease activity. Interestingly, the order Bacillales id.1674 was associated with a reduced risk of EA. This finding aligns with suggestions that *Bacillus* species can alleviate enterococcal spondylitis (“kinky back”) in poultry (38). However, we observed an increased risk of AS associated with the order Bacillales id.1674, suggesting that the same bacterium may exert opposing effects on different subtypes of SpA. This intriguing discrepancy warrants further investigation to better understand the complex interplay between Bacillales id.1674 and different SpA subtypes.

The potential link between SpA and gut microbiota has garnered increasing research attention in recent years (39–41). For instance, approximately 7% of patients with AS also have inflammatory bowel disease (IBD), while 10–50% of patients with IBD eventually develop SpA (42). HLA-B27 transgenic rats reared under germ-free conditions do not exhibit arthritic symptoms (43) until they are colonized by bacteria (44). Three hypotheses have been proposed to explain this, including the “arthritogenic peptide theory”, the “migration of mucosal cells to the joints theory”, and the “gut bacteria-driven theory” (45). Analyzing the correlation between SNPs in gut bacteria and susceptibility genes for SpA, such as HLA-B27 and IL-23/IL-17 pathway genes, is indeed a meaningful and valuable area of study (46). Several meta-analyses suggest that susceptibility genes, such as TNF-α polymorphisms (rs769178) (47), the SNPs (rs11209026, rs1004819, rs10489629, rs11465804, rs1343151, rs11209032, rs1495965, rs7517847, and rs2201841) of IL-23R (48), and the SNPs (rs27044, rs10050860, rs2287987, rs17482078, rs26653, rs30187, rs27037, rs27980, and rs27582) of endoplasmic reticulum aminopeptidase 1 (49), could influence the susceptibility to ankylosing spondylitis in the total population. In our study, it should be emphasized that the selected SNPs used in the MR analysis did not include the susceptibility gene SNPs mentioned in the aforementioned literature. Because our study did not specifically investigate the correlation between the selected SNPs and the susceptibility genes mentioned, we were unable to provide an exhaustive analysis of all the susceptibility genes involved in this matter. Further in-depth research is required to explore this relationship in future studies.

Our study represents the first attempt to establish a causal association between gut microbiota and SpA, including its subtypes, thus offering potential candidate bacteria for future functional investigations. Nevertheless, our study has several limitations. First, due to the stringent threshold (*p* < 5.0 × 10^−8^) required for instrumental variables, we adopted a relatively lenient threshold (*p* < 1.0 × 10^−5^) to screen for instrumental variables. This may have introduced some potential bias. Second, the study population is predominantly of European descent, and therefore the findings cannot be generalized to other ethnicities. Third, the EA subtype of SpA had a relatively small number of cases, based on its strict definition criteria. Consequently, future analyses using GWAS summary data from larger sample sizes are required to enhance confidence in our results. Fourth, current research on gut microbiota mainly focuses on bacteria; however, other types of gut microorganisms may also contribute significantly. In our study, we employed the MR-Egger intercept to detect pleiotropy. While these methods enhance the reliability of causal relationships in MR analysis, they do not conclusively rule out the presence of pleiotropy, as mentioned in our manuscript. Therefore, we recognize the significance of accounting for other biases and limitations in MR analysis and interpreting the findings with caution.

## 5 Conclusion

Through MR analysis, we conducted a comprehensive investigation of the causal association of 211 gut microbiota taxa on SpA. We ultimately identified 23 causal effects, including 22 nominal and one strong causal relationship. Specifically, the class Actinobacteria id.419 showed a significant association with an increased risk of AS. Our findings provide potential biomarkers and therapeutic targets for the progression of SpA.

## Data availability statement

The datasets presented in this study can be found in online repositories. The names of the repository/repositories and accession number(s) can be found in the article/**Supplementary Material**. Individual-level data was obtained from the FinnGen biobank (https://finngen.gitbook.io/documentation).

## Ethics statement

The requirement of ethical approval was waived by The ethics committee of the Hainan Branch of People’s Liberation Army General Hospital for the studies on humans because Ethical approval is not required for this study, as all summary-level data has already obtained ethical approval in previous GWAS studies. The studies were conducted in accordance with the local legislation and institutional requirements. Written informed consent for participation was not required from the participants or the participants’ legal guardians/next of kin in accordance with the national legislation and institutional requirements. The human samples used in this study were acquired from gifted from another research group.

## Author contributions

JT: Writing – original draft, Data curation, Funding acquisition, Resources. SM: Data curation, Writing – original draft. LF: Data curation, Writing – review & editing. SF: Writing – review & editing. XL: Writing – review & editing, Formal Analysis, Writing – original draft.
