# Supplementary material for: Causal association of gut microbiota on spondyloarthritis and its subtypes: a Mendelian randomization analysis
Source: Front Immunol. 2024 Feb 8;15:1284466. doi: 10.3389/fimmu.2024.1284466 (PMC10883304; doi:10.3389/fimmu.2024.1284466)
Supplement: Supplementary file 4 [file DataSheet_1.docx]

Supplementary Material

Causal Association of Gut Microbiota on Spondyloarthritis and Its Subtypes: A Mendelian Randomization Analysis

Jun Tang ^1†^, Shiyan Mo^2†^,Lina Fan^2^, Shihui Fu^3*^, Xiaofei Liu ^2*^

^1^Experimental Teaching Management Center, Chongqing Medical University, Chongqing, China

^2^Department of Rheumatology and Immunology, Hainan Hospital of Chinese PLA General Hospital, Sanya, Hainan, China.

^3^Department of Cardiovascular, Hainan Hospital of Chinese PLA General Hospital, Sanya, Hainan, China.

^†^These authors have contributed equally to this work and share first authorship

* Correspondence:
Shihui Fu

E-mail:xiaoxiao0915@126.com

Xiaofei Liu

E-mail: [xiaofeiliu1986@163.com](mailto:xiaofeiliu1986@163.com)

## Supplementary Figures

**Figure S1.** **Scatter plots for causal effects of gut microbiota on Ankylosing spondylitis risk using five MR methods.**A)class Actinobacteria id.419;B) family Lactobacillaceae id.1836;C) family Rikenellaceae id.967;D) genus Anaerotruncus id.2054;E) genus Enterorhabdus id.820;F) genus Howardella id.2000;G) genus Oscillibacter id.2063;H) genus Ruminococcaceae NK4A214 group id.11358; I) order Bacillales id.1674.Abbreviations:MR,Mendelian Randomization.

**
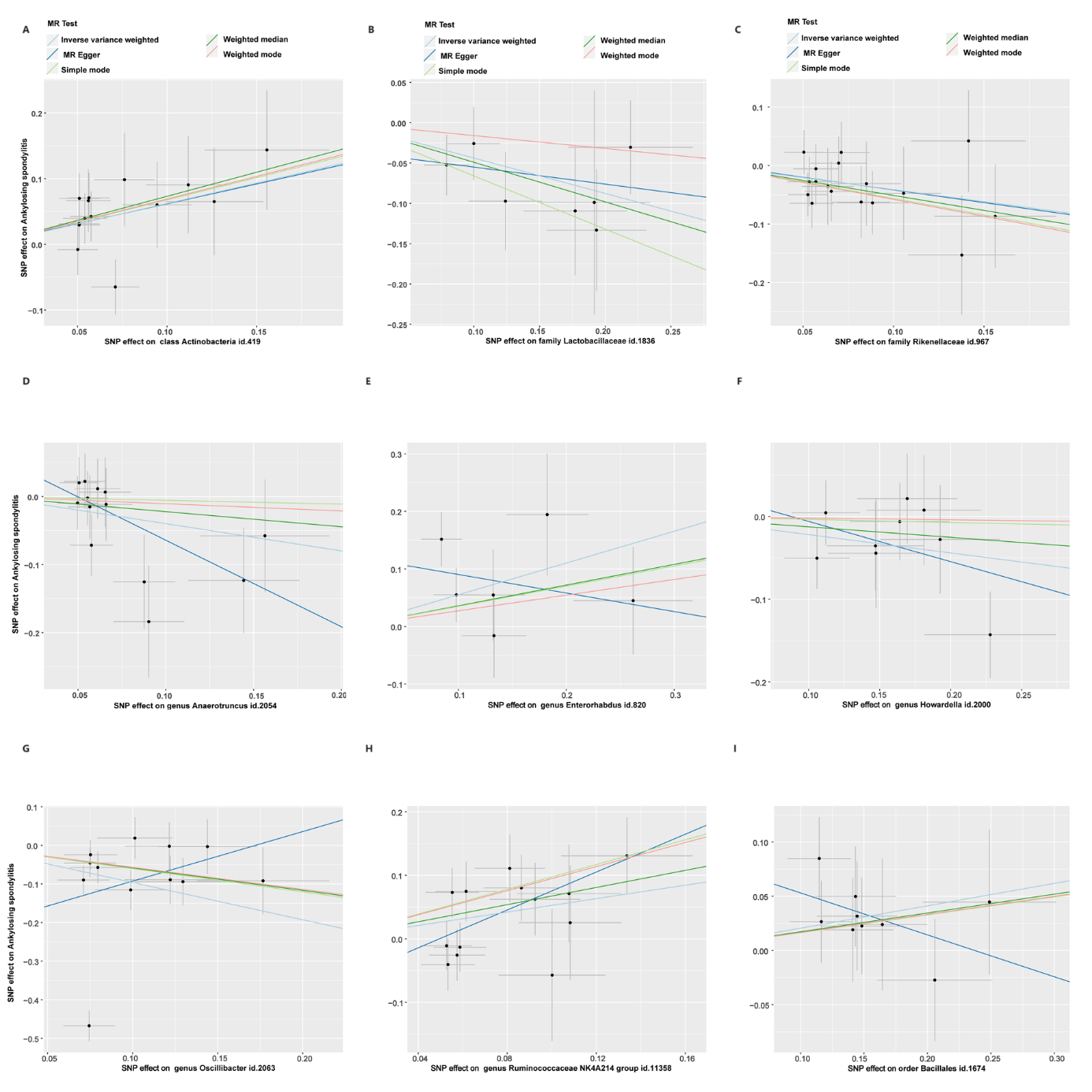
**

**Figure S2. Scatter plots for causal effects of gut microbiota on Arthropathic psoriasis risk using five MR methods.**A)class Verrucomicrobiae id.4029;B) family Rikenellaceae id.967; C) family Verrucomicrobiaceae id.4036; D) genus Akkermansia id.4037; E) genus Coprococcus1 id.11301; F) genus Lactococcus id.1851; G) genus Odoribacter id.952;H) order Verrucomicrobiales id.4030.Abbreviations: MR,Mendelian Randomization.

**
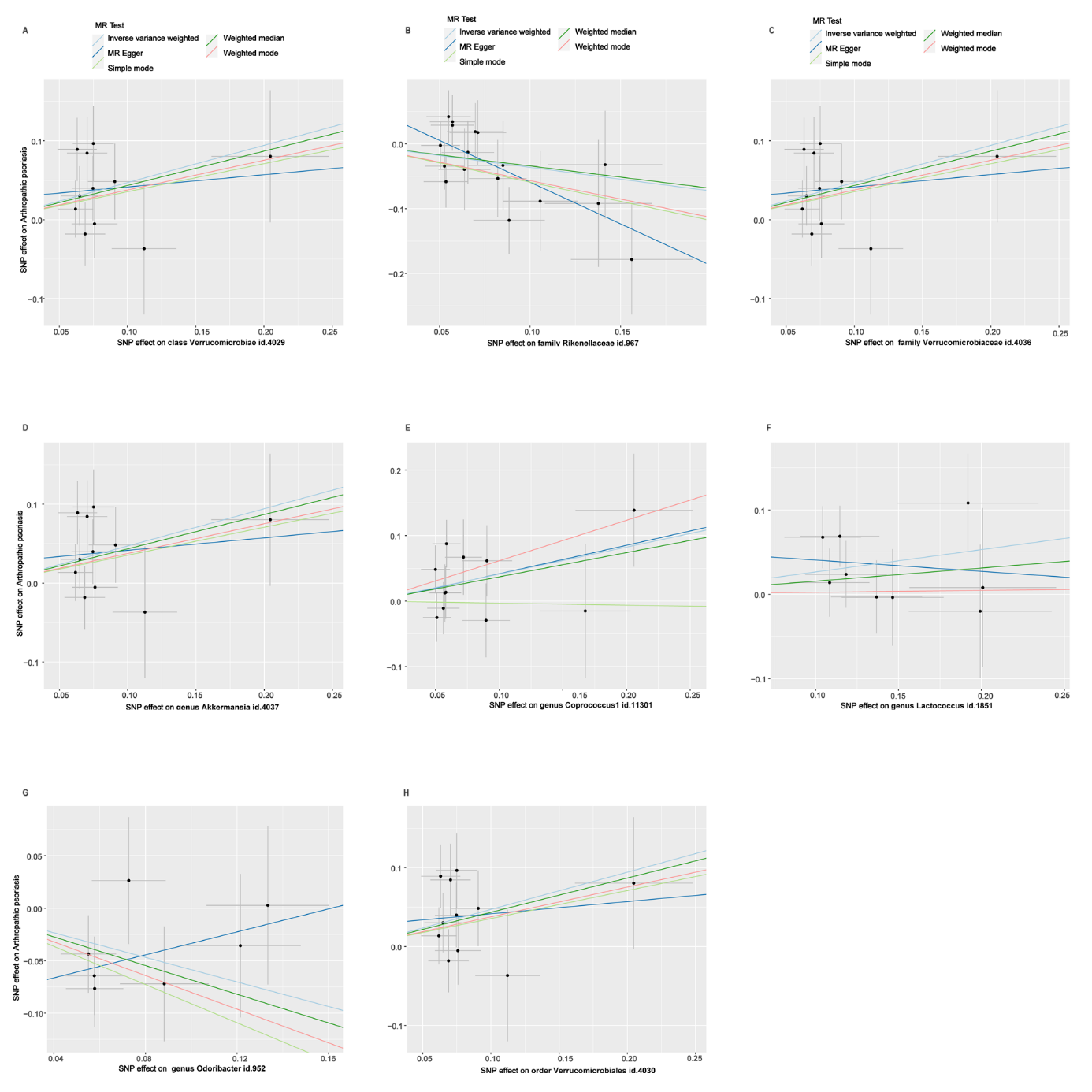
**

**Figure S3. Scatter plots for causal effects of gut microbiota on Enteropathic arthropathies risk using five MR methods.**A) genus Anaerostipes id.1991;B) genus Lachnospiraceae FCS020 group id.11314 ;C) genus Olsenella id.822; D) genus Parabacteroides id.954; E) genus Ruminococcaceae UCG013 id.11370; F) order Bacillales id.1674.Abbreviations: MR,Mendelian Randomization.

**
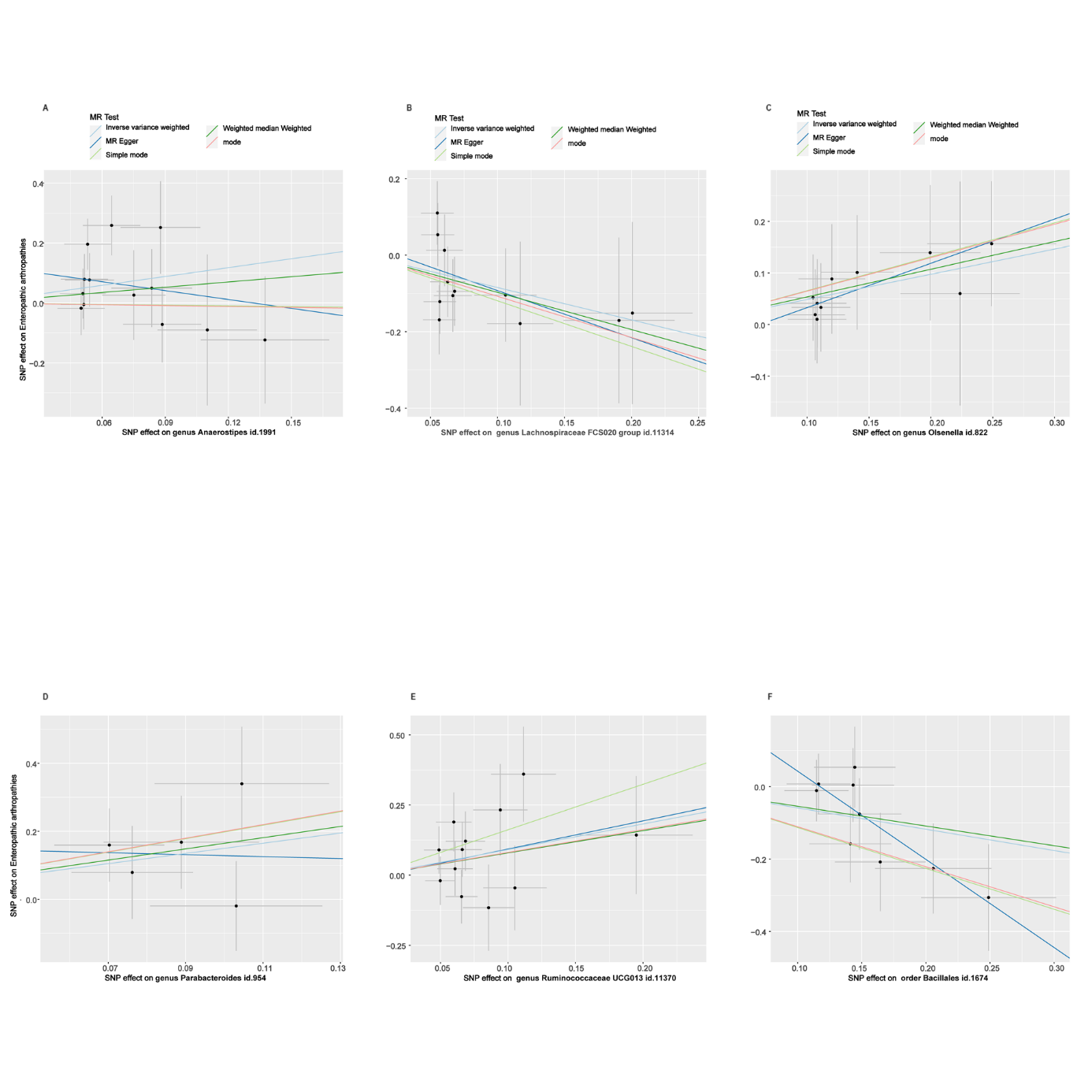
**

**Figure S4. Forest plots for causal effects of gut microbiota on Ankylosing spondylitis risk with individual SNPs.** A)class Actinobacteria id.419;B) family Lactobacillaceae id.1836;C) family Rikenellaceae id.967;D) genus Anaerotruncus id.2054;E) genus Enterorhabdus id.820;F) genus Howardella id.2000;G) genus Oscillibacter id.2063;H) genus Ruminococcaceae NK4A214 group id.11358; I) order Bacillales id.1674.Abbreviations:SNP,single-nucleotide polymorphism.

**
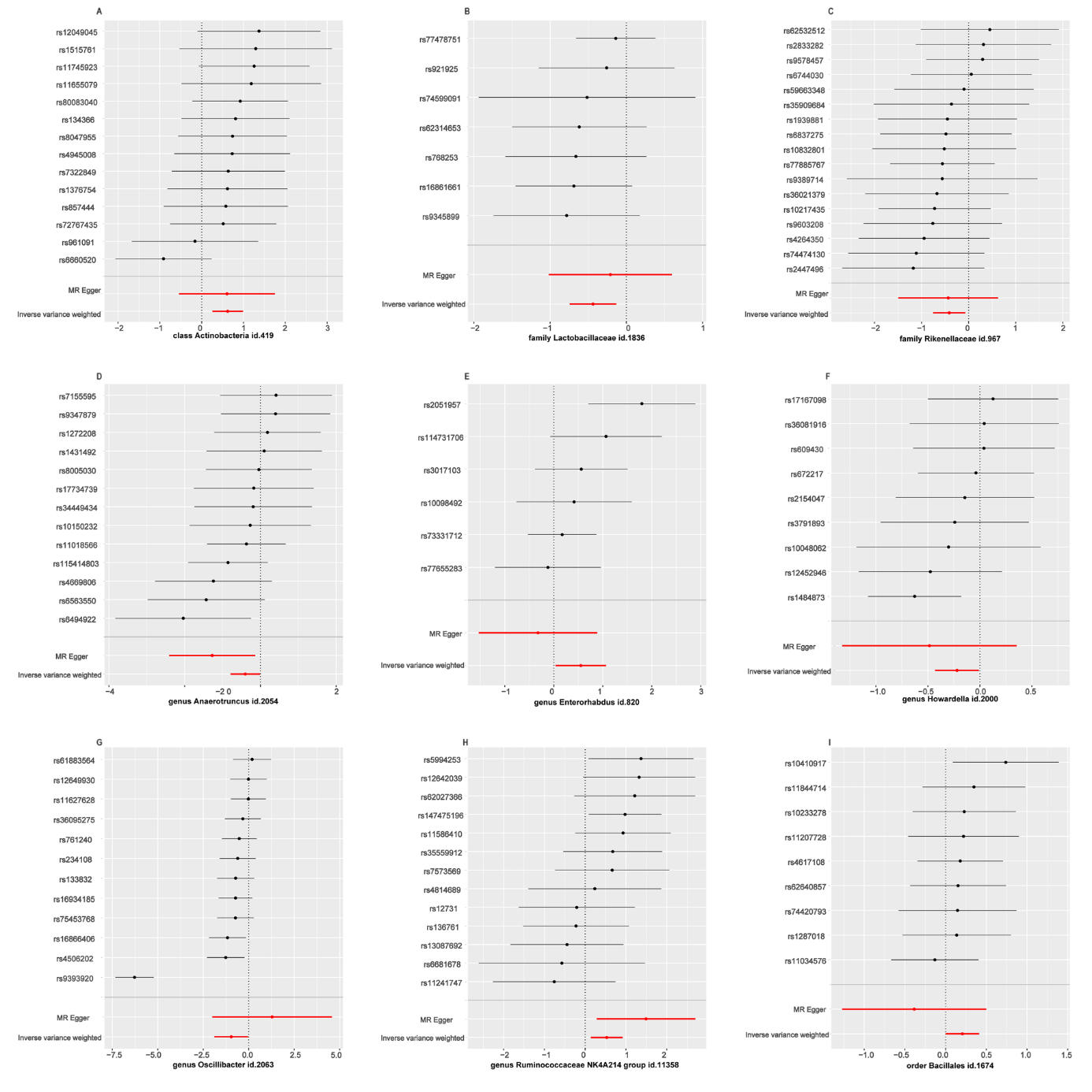
**

**Figure S5. Forest plots for causal effects of gut microbiota on Arthropathic psoriasis risk with individual SNPs.** A)class Verrucomicrobiae id.4029;B) family Rikenellaceae id.967; C) family Verrucomicrobiaceae id.4036; D) genus Akkermansia id.4037; E) genus Coprococcus1 id.11301; F) genus Lactococcus id.1851; G) genus Odoribacter id.952;H) order Verrucomicrobiales id.4030.Abbreviations: SNP,single-nucleotide polymorphism.

**
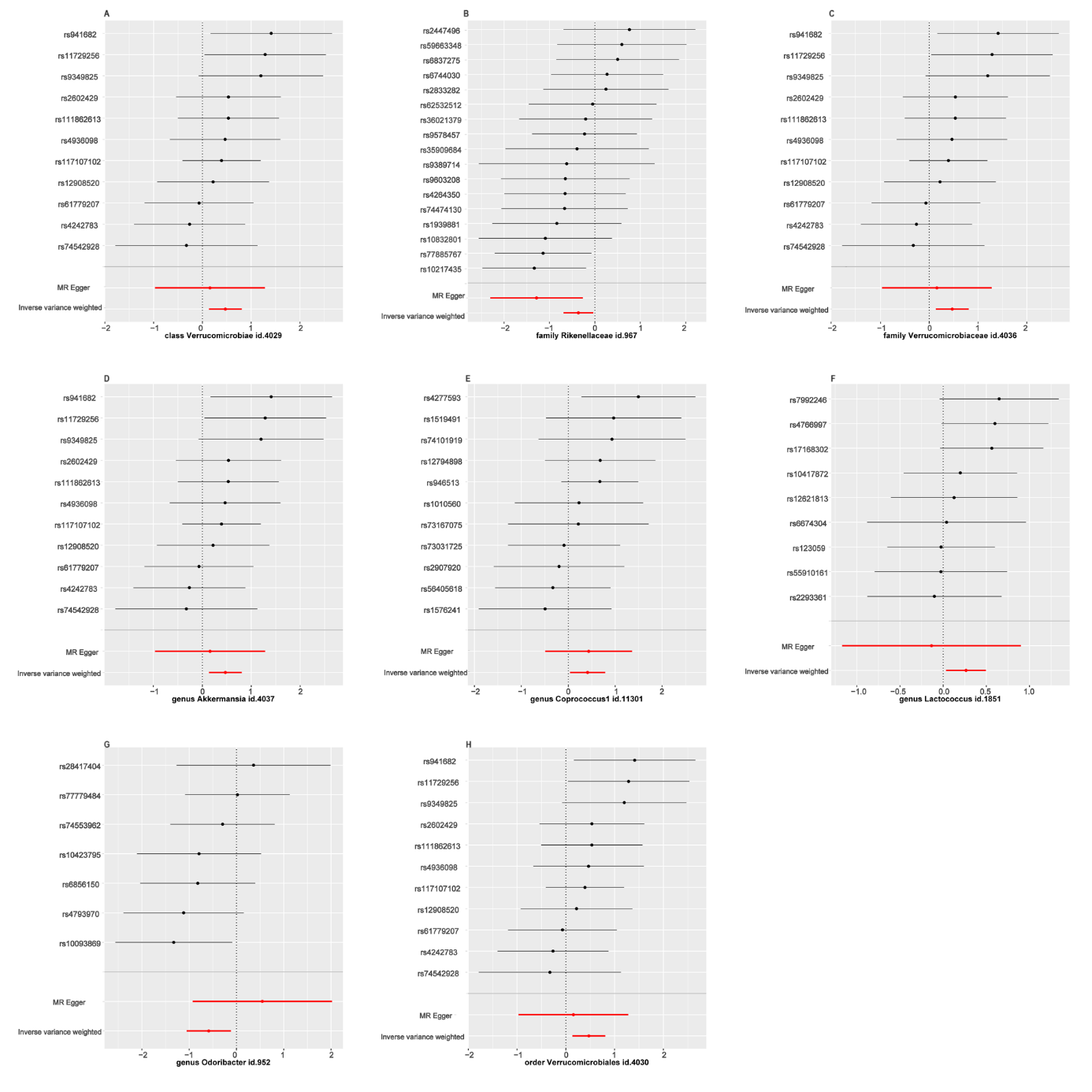
**

**Figure S6. Forest plots for causal effects of gut microbiota on** **Enteropathic arthropathies risk with individual SNPs.** A) genus Anaerostipes id.1991;B) genus Lachnospiraceae FCS020 group id.11314 ;C) genus Olsenella id.822; D) genus Parabacteroides id.954; E) genus Ruminococcaceae UCG013 id.11370; F) order Bacillales id.1674.Abbreviations: SNP,single-nucleotide polymorphism.

**
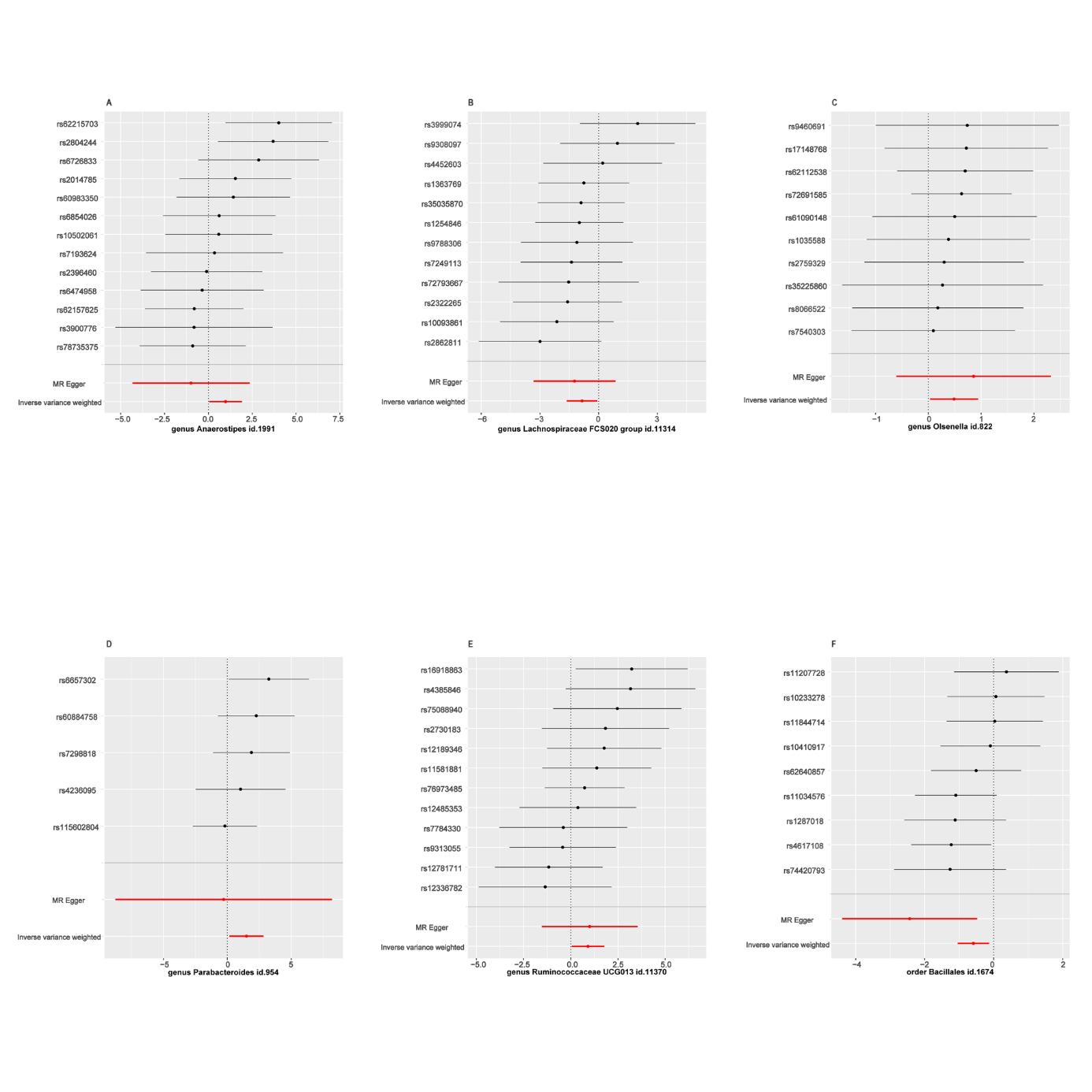
**

**Figure S7.Plots for "leave-one-out" analysis for causal effect ofgutmicrobiota on** **Ankylosing spondylitis risk.** A)class Actinobacteria id.419;B) family Lactobacillaceae id.1836;C) family Rikenellaceae id.967;D) genus Anaerotruncus id.2054;E) genus Enterorhabdus id.820;F) genus Howardella id.2000;G) genus Oscillibacter id.2063;H) genus Ruminococcaceae NK4A214 group id.11358; I) order Bacillales id.1674.

**
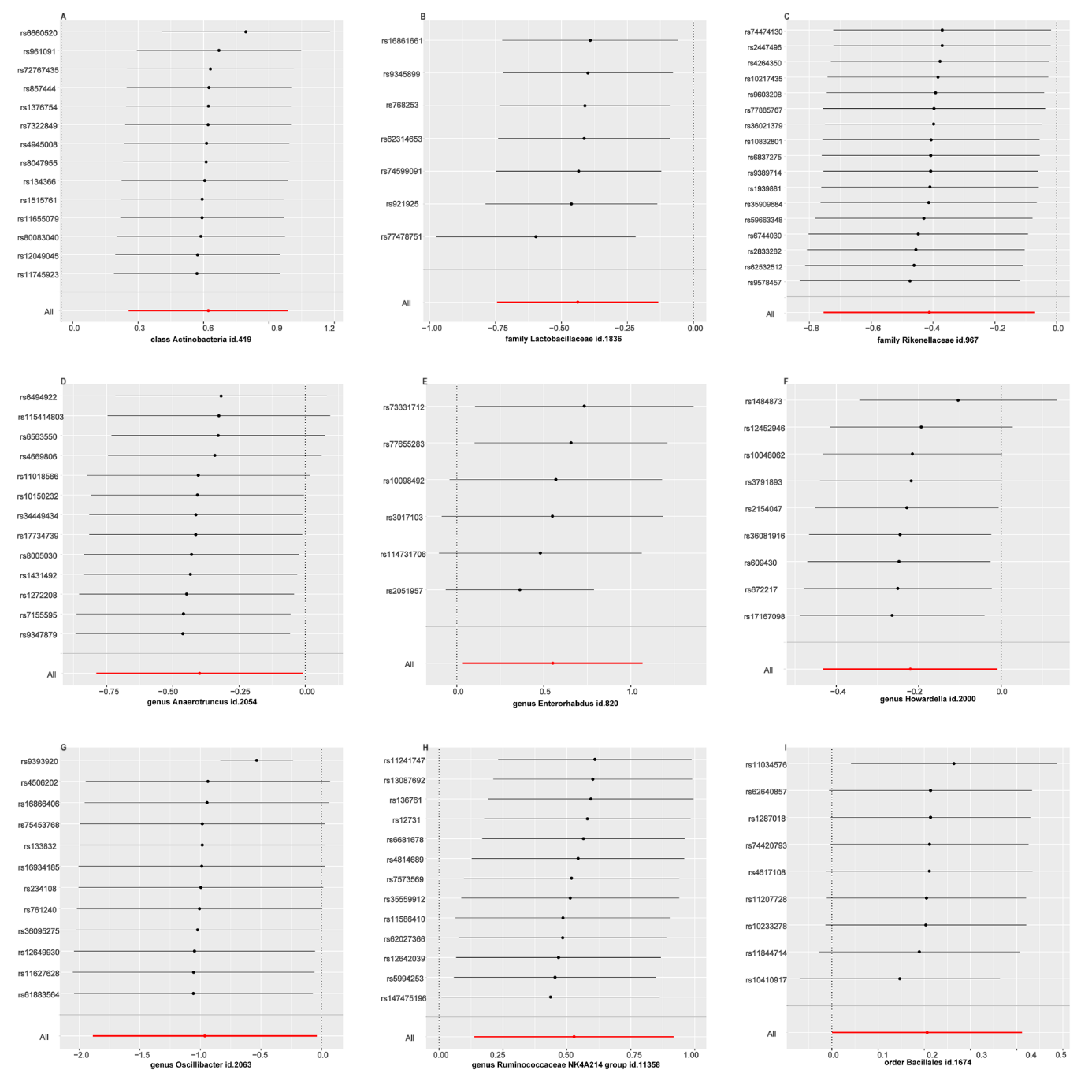
**

**Figure S8. Plots for "leave-one-out" analysis for causal effect of gut microbiota on** **Arthropathic psoriasis risk.** A)class Verrucomicrobiae id.4029;B) family Rikenellaceae id.967; C) family Verrucomicrobiaceae id.4036; D) genus Akkermansia id.4037; E) genus Coprococcus1 id.11301; F) genus Lactococcus id.1851; G) genus Odoribacter id.952;H) order Verrucomicrobiales id.4030.

**
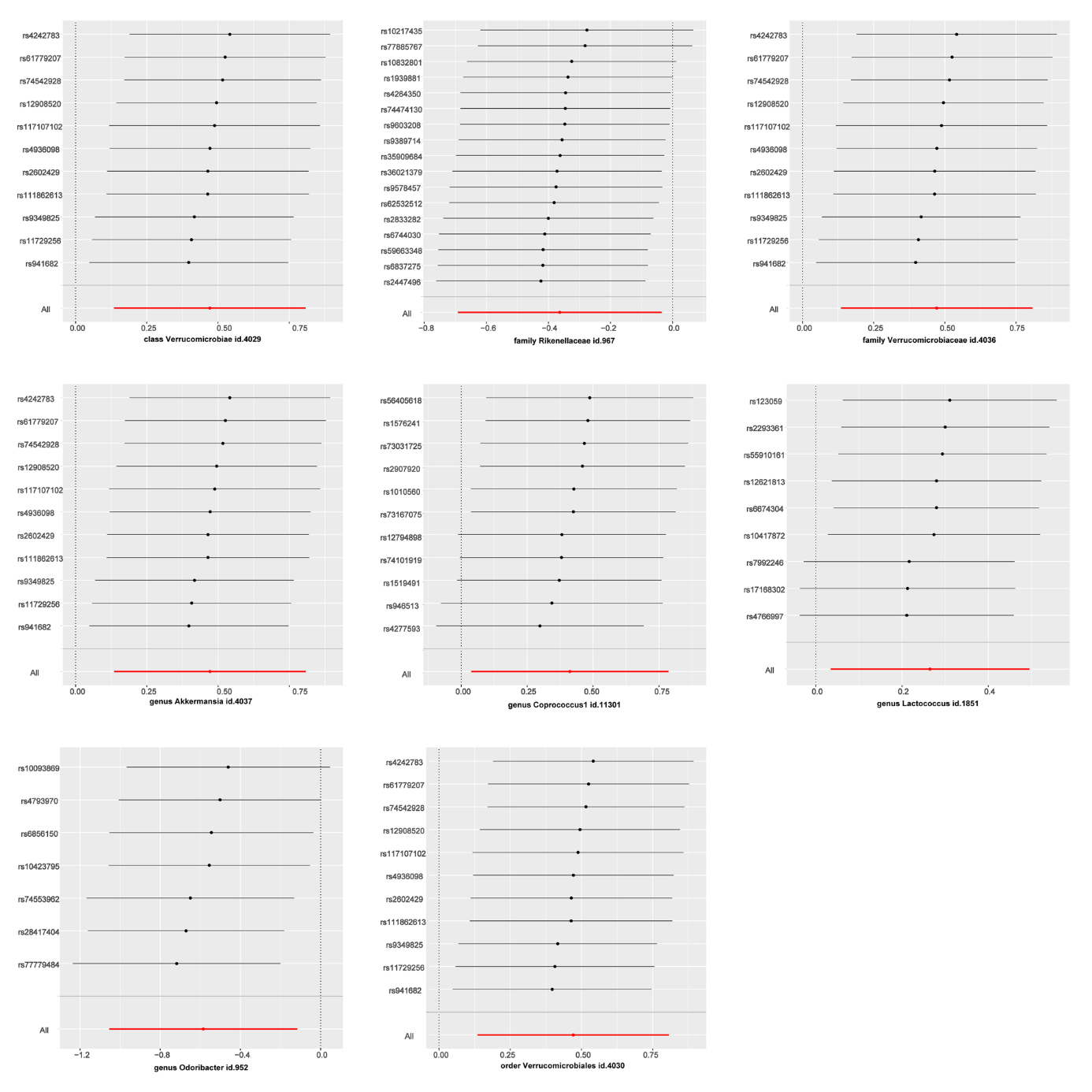
**

**Figure S9. Plots for "leave-one-out" analysis for causal effect of gut microbiota on Enteropathic arthropathies risk.** A) genus Anaerostipes id.1991;B) genus Lachnospiraceae FCS020 group id.11314 ;C) genus Olsenella id.822; D) genus Parabacteroides id.954; E) genus Ruminococcaceae UCG013 id.11370; F) order Bacillales id.1674.

**
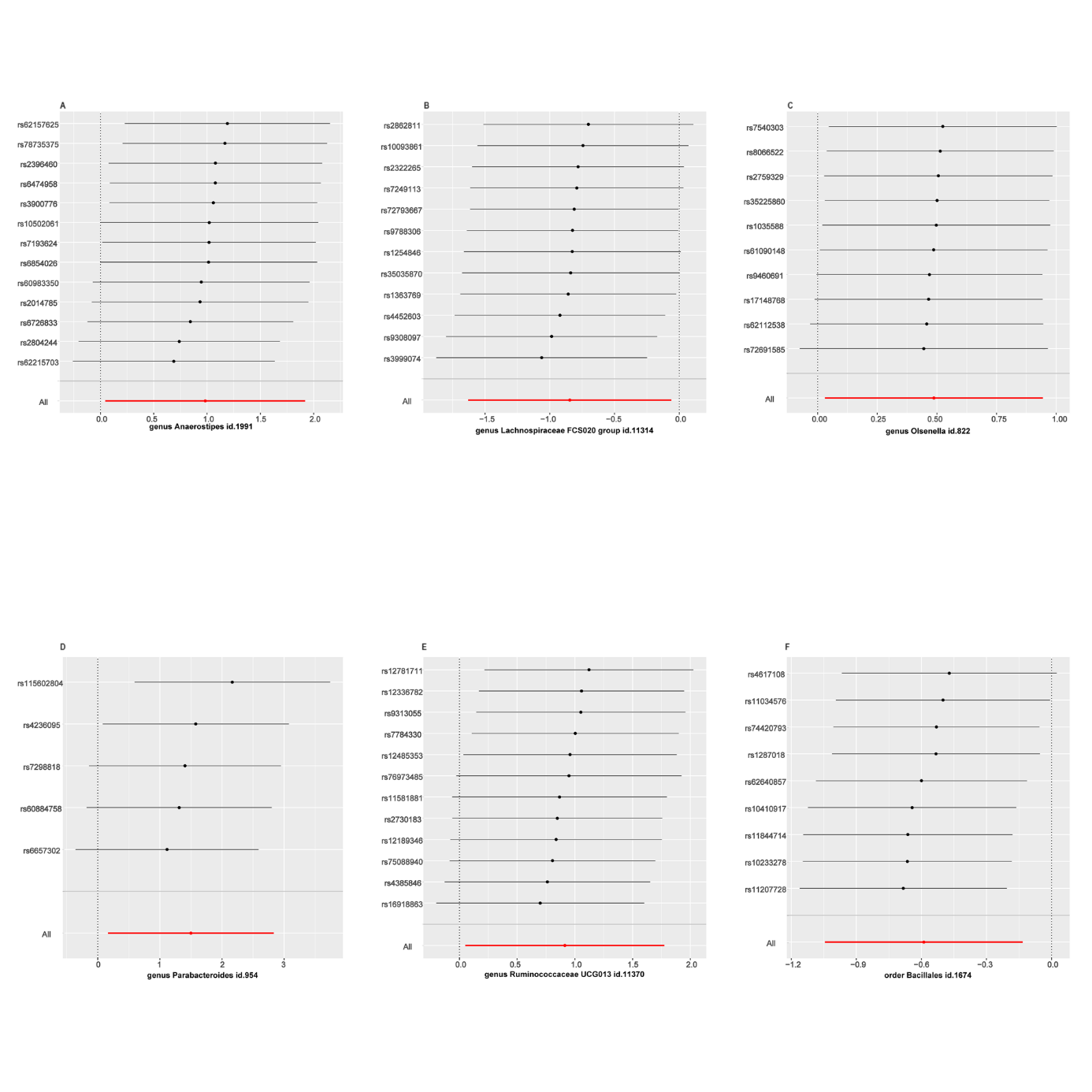
**
